# Disease and patient characteristics in NP-C patients: findings from an international disease registry

**DOI:** 10.1186/1750-1172-8-12

**Published:** 2013-01-16

**Authors:** Marc C Patterson, Eugen Mengel, Frits A Wijburg, Audrey Muller, Barbara Schwierin, Harir Drevon, Marie T Vanier, Mercé Pineda

**Affiliations:** 1 Mayo Clinic, Rochester, MN, USA; 2 Villa Metabolica, ZKJM, MC, University of Mainz, Mainz, Germany; 3 Academic Medical Centre, University of Amsterdam, Amsterdam, The Netherlands; 4 Actelion Pharmaceuticals Ltd, Allschwil, Switzerland; 5 Numerus Ltd., Wokingham, UK; 6 INSERM Unit 820, Lyon, France; 7 Fundació Hospital Sant Joan de Déu, Barcelona, Spain; 8 Department of Neurology, Mayo Clinic, 200 First Street SW, Rochester, MN, 55905, USA

**Keywords:** Niemann-Pick disease type C, Diagnosis, Symptoms, Neurological, Hepatomegaly, Splenomegaly, Cholestasis, Vertical supranuclear palsy

## Abstract

**Background:**

Niemann-Pick disease type C (NP-C) is a rare neurovisceral disease characterized by progressive neurodegeneration and premature death. We report data recorded at enrolment in an ongoing international NP-C registry initiated in September 2009 to describe disease natural history, clinical course and treatment experience of NP-C patients in clinical practice settings.

**Methods:**

The NPC Registry is a prospective observational cohort study. Participating sites are encouraged to evaluate all consecutive patients with a confirmed diagnosis of NP-C, regardless of their treatment status. All patients undergo clinical assessments and medical care as determined by their physicians. Data are collected through a secure internet-based data collection system.

**Results:**

As of 19^th^ March, 2012, 163 patients have been enrolled in centres across 14 European countries, Australia, Brazil and Canada. The mean (SD) age at enrolment was 19.6 (13.0) years. In general there was a long lag time between the mean (SD) age at neurological onset (10.9 (9.8) years) and age at diagnosis (15.0 (12.2) years). Among all enrolled patients, 107 were diagnosed based on combined genetic testing and filipin staining. Sixteen (11%) out of 145 patients with available age-at-neurological-onset data had early-infantile neurological onset, 45 (31%) had late-infantile onset; 45 (31%) had juvenile onset and 39 (27%) had adolescent/adult onset. The frequencies of neonatal jaundice, hepatomegaly and/or splenomegaly during infancy were greatest among early-infantile patients, and decreased with increasing age at neurological onset. The most frequent neurological manifestations were: ataxia (70%), vertical supranuclear gaze palsy (VSGP; 70%), dysarthria (66%), cognitive impairment (62%), dysphagia (52%). There were no notable differences in composite NP-C disability scores between age-at-neurological-onset groups. Miglustat therapy at enrolment was recorded in 117/163 (72%) patients.

**Conclusions:**

Approximately two-thirds of this NP-C cohort had infantile or juvenile onset of neurological manifestations, while the remaining third presented in adolescence or adulthood. While systemic symptoms were most common among patients with early-childhood onset disease, they were also common among patients with adolescent/adult onset. The profiles of neurological manifestations in this Registry were in line with previous publications.

## Background

Niemann-Pick disease type C (NP-C) is a rare neurovisceral disease characterized by progressive neurodegeneration and premature death, with an estimated incidence at diagnosis of 1:100,000–120,000 live births [1,2]. NP-C is caused by autosomal recessive mutations in the *NPC1* gene (in 95% of cases) or the *NPC2* gene, leading to severely impaired intracellular lipid transport and accumulation of unesterified cholesterol, sphingosine and a range of glycosphingolipids in various tissues, including the brain [3,4].

Advances in knowledge regarding the underlying biochemical disease pathways in NP-C over the last 20 years, such as the discovery of cholesterol storage abnormalities and characterisation of causal gene mutations, have led to increasing awareness of the disease among physicians and a large number of new diagnoses. In turn, the broad clinical heterogeneity of NP-C has become increasingly recognised based on several observational studies [2,5-9].

Clinical presentations of NP-C feature a range of systemic and neurological signs that are not specific to the disease, arise at different ages, and progress at different rates [1,2,5,6,9,10]. Very early-onset patients are often diagnosed based on isolated systemic manifestations [11,12], but patients most often present during childhood with one or more neurological manifestations such as saccadic eye movement abnormalities, cerebellar ataxia, learning problems and clumsiness [1,9,10]. Increasing numbers of patients with adolescent/adult onset of neurological disease are also being detected and diagnosed [13-15].

Recent large cohort studies have shown that categorization of patients according to age at onset of neurological manifestations is useful for the evaluation of disease course and responses to therapy, and helps in clinical disease management and genetic counselling [1,2,6,10,16]. In addition, the establishment of disease-specific disability scales and their application in a number of observational studies has allowed more objective, semi-quantitative assessments of neurological disease progression and response to therapy [6,16-18].

A variety of symptomatic treatments can alleviate the neurological manifestations of NP-C [19], and the appropriate application of such therapies can have an important influence on patient quality of life [10]. Miglustat (Zavesca®; Actelion Pharmaceuticals Ltd) is currently the only disease-specific therapy for the treatment of progressive neurological manifestations in children and adults with NP-C [20]. Miglustat was approved in Europe in 2009, and has since been approved in a number of other countries. As a result, clinical experience with this drug is increasing [7,8,16,21].

Here, we report data from an ongoing international NP-C registry, which was initiated to describe the natural history, disease course, clinical outcomes and treatment experience of NP-C patients in clinical practice settings. The NPC Registry enrols patients regardless of their treatment status, although a large proportion is receiving miglustat. As such, the Registry forms part of the post-marketing surveillance for miglustat. Patient and disease characteristics at enrolment in all patients included in the Registry as of 19^th^ March, 2012 are summarised.

## Methods

### Study design and population

The NPC Registry is an international, prospective observational cohort study. All patients with a confirmed diagnosis of NP-C are eligible for entry regardless of their treatment status. Participating sites are encouraged to evaluate all consecutive NP-C patients (both incident and prevalent) attending outpatient and/or inpatient visits.

Patients included in the Registry undergo clinical assessments and medical care as determined by their physicians.

### Data collection and assessments

Data on the demographics, diagnosis, disease characteristics and treatment of patients from clinical practice settings are entered through a secure, internet-based data collection system. Data are only recorded from visits that take place after all necessary study-specific and site-specific approvals as well as written informed consent were received. Before entering any visit data, written informed consent was obtained from all patients (including children) and/or their legal guardians.

Data collected include information from assessments that are routinely performed for NP-C patients in clinical practice: demographics, diagnostic tests and findings, medications (including miglustat exposure), non-drug therapies, growth characteristics, neurological manifestations, functional disability. Physicians are also encouraged to enter data on the medical history, general physical status of patients and findings from other assessments performed: organ volumes, ears and eyes examinations, laboratory findings, and neuroimaging.

Patient functional disability status is evaluated using a disease-specific disability scale that assesses four key domains of neurological disease in NP-C [6,18]. Briefly, ambulation is assigned a rating on a five-point scale ranging from normal to wheelchair-bound. Manipulation is rated on a four-point scale from normal to severe dysmetria/dystonia. Language function is rated on a five-point scale from normal to absence of communication, and swallowing is rated a four-point scale from normal to gastric button feeding. Based on these ratings, each functional domain was assigned a score ranging from 0 (best) to 1 (worst) to provide equal weighting across all domains.

An independent Scientific Committee advises on medical and scientific aspects of the Registry. For data quality assurance, the data collection system keeps an audit trail of all entries and changes, and performs data checks of both data range and plausibility. In addition, the study database is also periodically reviewed for missing data and incomplete information.

### Data analysis

This analysis summarises patient data recorded at enrolment, which includes information from clinical assessments as outlined above, as well as relevant data from patient histories based on their medical records. Age at diagnosis, age at neurological disease onset, time since diagnosis, treatment exposure time and disease severity are also provided. Patient ages at diagnosis are calculated based on the date at which the first positive genetic and/or positive filipin staining test results were reported.

NP-C disability scale analyses were based on scores for all four functional domains as well as a composite score, which is calculated as the mean of all four domain scores [18]. Disability scale data and other relevant patient/disease characteristics were stratified by age at onset of neurological manifestations according to previously published age categories: early-infantile onset (<2 years), late-infantile onset (2 to <6 years), juvenile onset (6 to <15 years), and adolescent/adult onset (≥15 years) [1,2,6,10]. Due to the limited number of cases (n = 3), pre/peri-natal patients (aged between 0 and 3 months) were merged with the early-infantile onset category.

Analyses of data at enrolment are purely descriptive in nature. Continuous variables are summarised using descriptive statistics including mean, standard deviation (SD), median, range and 95% confidence intervals (CI) of the mean. Categorical variables are summarised using counts and percentages. By nature, this is an observed cases analysis, with all summary statistics and percentages calculated relative to number of patients with available data. In the following sections, the denominators for analysis were the numbers of patients with the corresponding data available; different parameters have different denominators.

## Results

### Demographics

**Figure 1:**
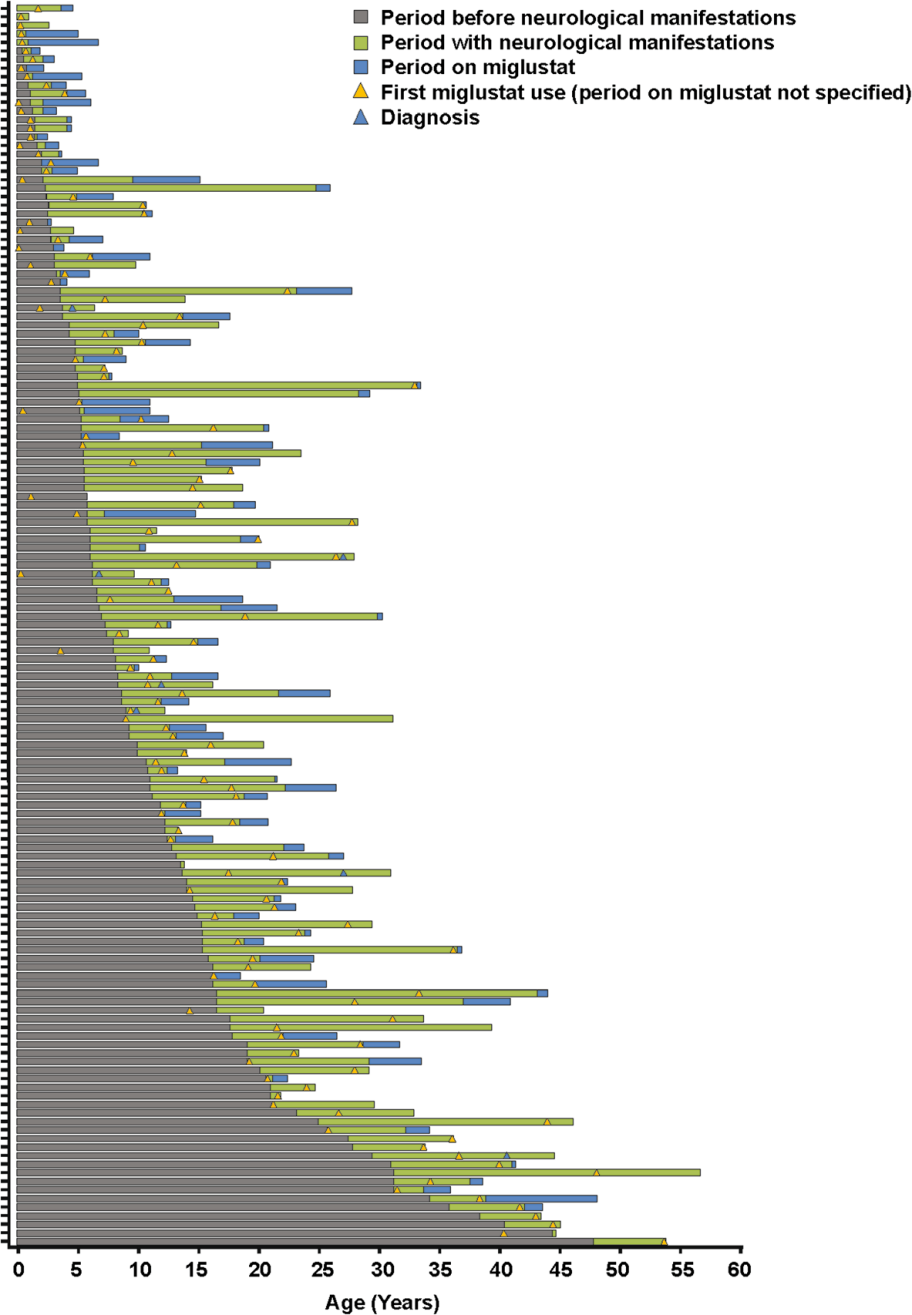
Overview of patient and disease characteristics.

A total of 163 patients have been enrolled as of 19^th^ March, 2012. Patients have been enrolled from centres in 14 European countries, Australia, Brazil and Canada. Table 1 and Figure 1 provide an overview of patient and disease characteristics and of relevant age milestones.

**Table 1:** Demographics and age at diagnosis of all patients at enrolment

**Characteristic**	**n (%)**	**Mean (SD)**	**Median (range)**
**Overall patient population (n = 163)**			
Gender, male: female	84 (51.5): 79 (48.5)	–	–
Age at enrolment (yrs)	163	19.6 (13.0)	17.1 (0.9–64.1)
**Patients with neurological manifestations (n =146)**			
Age at neurological onset (yrs):	145*	10.9 (9.8)	7.4 (0–48.0)
Early infantile (<2 yrs)	16 (11)	0.8 (0.6)	1.0 (0–1.6)
Late infantile (2 – <6 yrs)	45 (31)	4.2 (1.3)	4.5 (2.0–6.0)
Juvenile (6 – <15 yrs)	45 (31)	9.7 (2.8)	9.2 (6.0–14.8)
Adolescent/adult (≥15 yrs)	39 (27)	24.0 (8.9)	20.9 (15.0–48.0)
Age at diagnosis (yrs):	140^†^	15.0 (12.2)	12.8 (0.1–53.9)
Early infantile (<2 yrs)	16 (12)^‡^	1.0 (1.0)	0.8 (0.1–4.0)
Late infantile (2 – <6 yrs)	43 (31)^‡^	8.2 (7.4)	6.1 (0.1–33.1)
Juvenile (6 – <15 yrs)	41 (30)^‡^	13.8 (5.0)	13.0 (0.4–26.6)
Adolescent/adult (≥15 yrs)	39 (28)^‡^	29.7 (10.0)	28.1 (14.4–53.9)
**Patients with no neurological manifestations (n = 5)**			
Age at enrolment (yrs)	5 (3)	15.9 (14.6)	11.4 (4.3–40.9)
Age at diagnosis (yrs)	4 (2)^#^	12.4 (16.8)	5.3 (1.9–37.3)
History of systemic symptoms:			
Neonatal jaundice, present	2 (40)	–	–
Hepatomegaly during infancy, present	1 (20)	–	–
Splenomegaly during infancy, present	2 (40)	–	–

*One patient had missing age at neurological onset, five had no neurological manifestations and 12 had no available information regarding neurological manifestations. ^†^Six patients with missing age at diagnosis data; ^‡^percentages calculated relative to total patients with available data for both age at diagnosis and age at neurological onset (N = 139); ^#^age at diagnosis data missing in one patient.

The mean (SD) age at enrolment was 19.6 (13.0) years. A total of 146 patients had neurological symptoms prior to or at enrolment. Among patients with available age at neurological onset data (N = 145), 16 (11%) had the early-infantile onset form, 45 (31%) had late-infantile onset; 45 (31%) had juvenile onset and 39 (27%) had adolescent/adult onset. Three patients in the early infantile-onset group presented with perinatal hypotonia. Information regarding neurological manifestations was missing for 12 patients.

Forty-two patients had ‘psychiatric’ manifestations at enrolment. The proportion of adolescent/adult-onset patients among this subgroup was higher (16/40 [40%]) than those in the earlier-onset categories (3–30%).

Five patients had no neurological manifestations recorded prior to or at enrolment (Table 1). All five had a confirmed biochemical diagnosis based on filipin staining, among which three were also shown to carry *NPC1* mutations. All five patients had visceral involvement at enrolment; histories of neonatal jaundice, hepatomegaly and/or splenomegaly during infancy were recorded in two patients each.

One adult male patient (current age 43 years; enrolled at age 41 years), who displayed only hepatosplenomegaly at enrolment was diagnosed aged 37 years based on positive filipin staining. No genetic testing results were available and cholesterol esterification was not assessed. This patient did not have a history of visceral manifestations during infancy.

### Diagnosis

Among all 163 enrolled patients, 107 had an NP-C diagnosis established by combined genetic testing and filipin staining, 39 by genetic testing alone and 14 by filipin staining alone. Documentation related to diagnostic test results was missing for three patients.

Filipin staining was recorded as having been performed in 121 out of 140 patients with available data (86%). The filipin test gave positive results for 113 patients. Results were reported as negative in two patients, one with juvenile-onset (aged 6.9 years) and one with adolescent/adult-onset of neurological manifestations (aged 17.9 years), although both of these patients had disease-causing *NPC1* gene mutations. Filipin staining results were not known for six patients.

Gene mutation analysis was recorded as having been performed in 146 out of 154 patients with available data (95%). Of the 137 patients with available genetic results, 134 had *NPC1* mutations and three had *NPC2* mutations. Results from genetic testing were not known for nine patients.

Findings from LDL-induced cholesteryl ester formation assays were recorded in 63 out of 120 patients with available data (53%). Among these, 56 patients had a reduced rate of cholesteryl ester formation. Five patients had a normal rate: one patient (aged 5 years) had missing age-at-onset data but did have neurological manifestations at enrolment; one (aged 9 years) had no neurological manifestations; two had juvenile neurological onset; one had adult neurological onset.

### Patient age milestones

In general there was a long lag time between the mean (SD) age at neurological onset (10.9 (9.8) years; range 0–48 years) and age at diagnosis (15.0 (12.2) years; range 0.1–53.9 years) (Table 1). This period appeared longer among adolescent/adult-onset patients compared with the early-, late- and juvenile-onset groups.

Eight out of 16 (50%) early-infantile onset patients were diagnosed with NP-C before the appearance of neurological manifestations. Most (72–95%) late-infantile, juvenile and adolescent/adult-onset patients were diagnosed after neurological onset.

### Systemic manifestations

**Figure 2:**
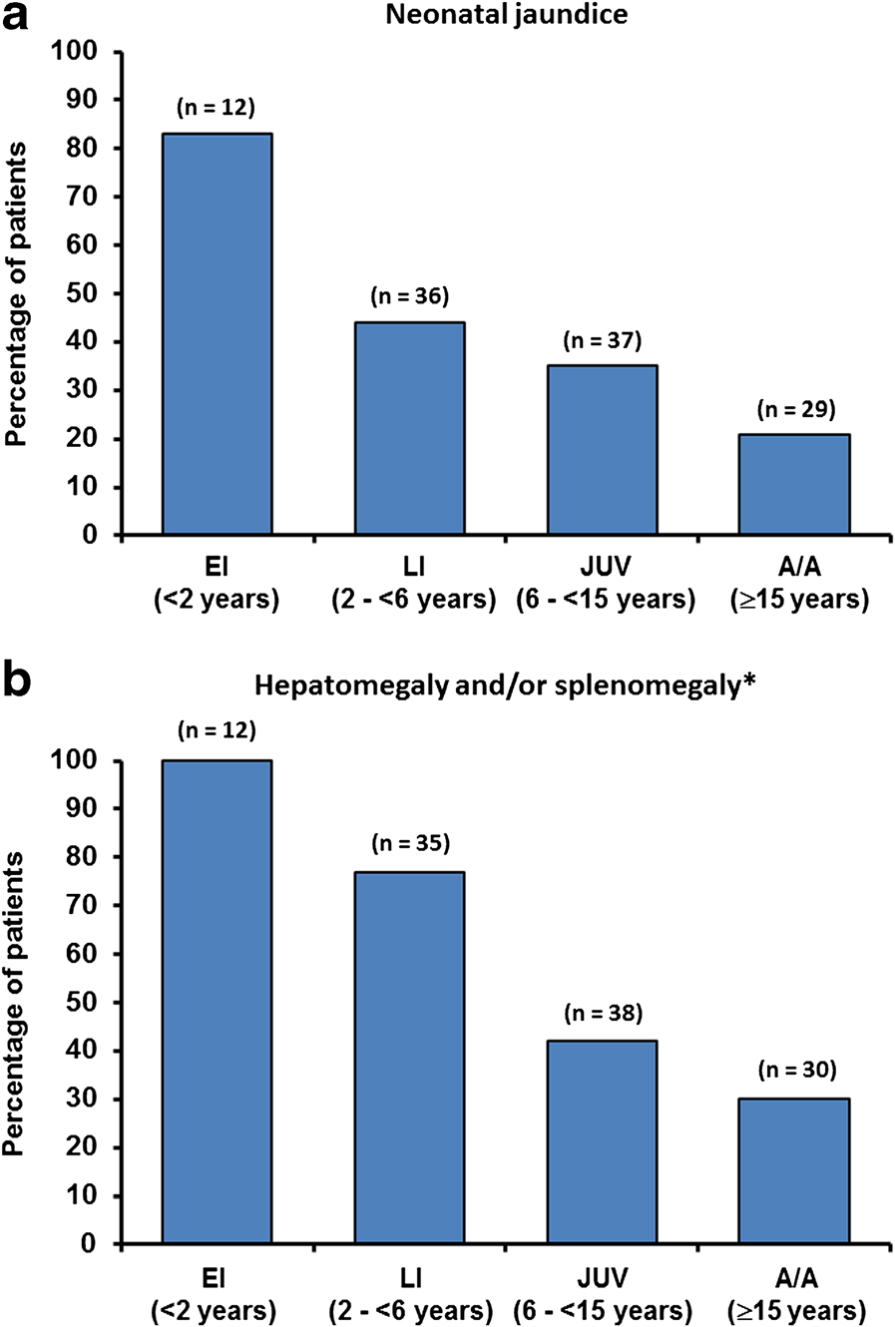
Percentage of patients with (a) neonatal jaundice and (b) hepatomegaly and/or splenomegaly [EI = early infantile, LI = late infantile, JUV = juvenile, A/A = adolescent/adult].

Data on patient histories of neonatal jaundice, hepatomegaly and/or splenomegaly during infancy are summarised in Figure 2. The overall proportions of these systemic manifestations were: neonatal jaundice during infancy (49/126; 39%); hepatomegaly during infancy (50/127; 39%); *s*plenomegaly (69/127; 54%). The prevalence of neonatal jaundice, hepatomegaly and/or splenomegaly during infancy was particularly high among early-infantile onset patients, and decreased with increasing age at neurological onset. Nevertheless, 21% of adolescent/adult-onset patients had a history of neonatal jaundice and 30% had hepatosplenomegaly during infancy.

Overall, a history of hepatic failure during the neonatal period was recorded in 7/120 (6%) patients. Hepatic failure was recorded as having resolved in six of these cases. Systemic outcome data were missing for the remaining patient.

### Neurological manifestations

Based on medical data recorded prior to patients’ enrolment in the Registry, the most frequent neurological manifestations among all those with available data (N = 138) were: ataxia (70%), vertical supranuclear gaze palsy (VSGP; 70%), dysarthria (66%), cognitive impairment (62%), dysphagia (52%), dystonia (46%), seizures (33%), and cataplexy (26%).

**Figure 3:**
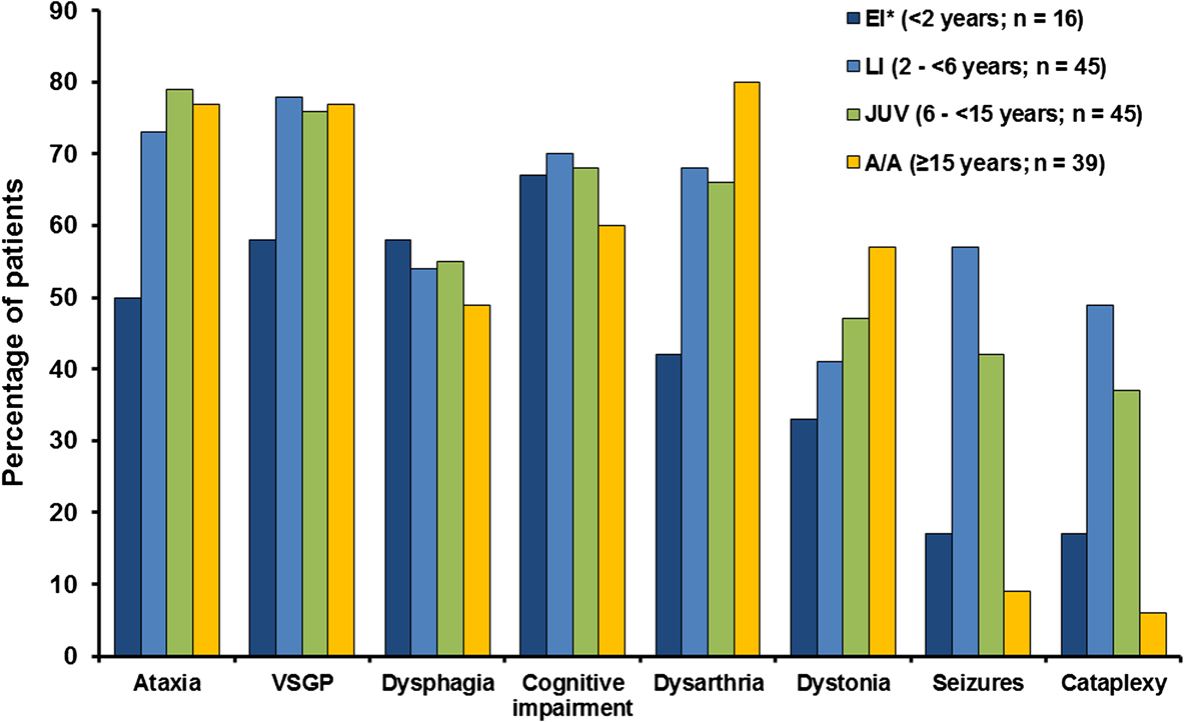
Neurological manifestations by age of onset [EI = early infantile, LI = late infantile, JUV = juvenile, A/A = adolescent/adult].

The profiles of neurological manifestations according to age-at-neurological-onset category are summarised in Figure 3. Ataxia and VSGP were both recorded in over 70% of late-infantile, juvenile and adolescent/adult-onset, and dysarthria in >60% of patients in these age categories. The prevalence of cognitive impairment (recoded as ‘delayed development’ in very young patients) was consistently high across all age groups (60–70%). Dysphagia was also a consistent finding in approximately 50% of all age categories. Seizures and cataplexy were more often recorded among late-infantile/juvenile onset patients (37–57%) compared with early-infantile onset (17%) and adolescent/adult-onset patients (6–9%).

### Disability score

**Figure 4:**
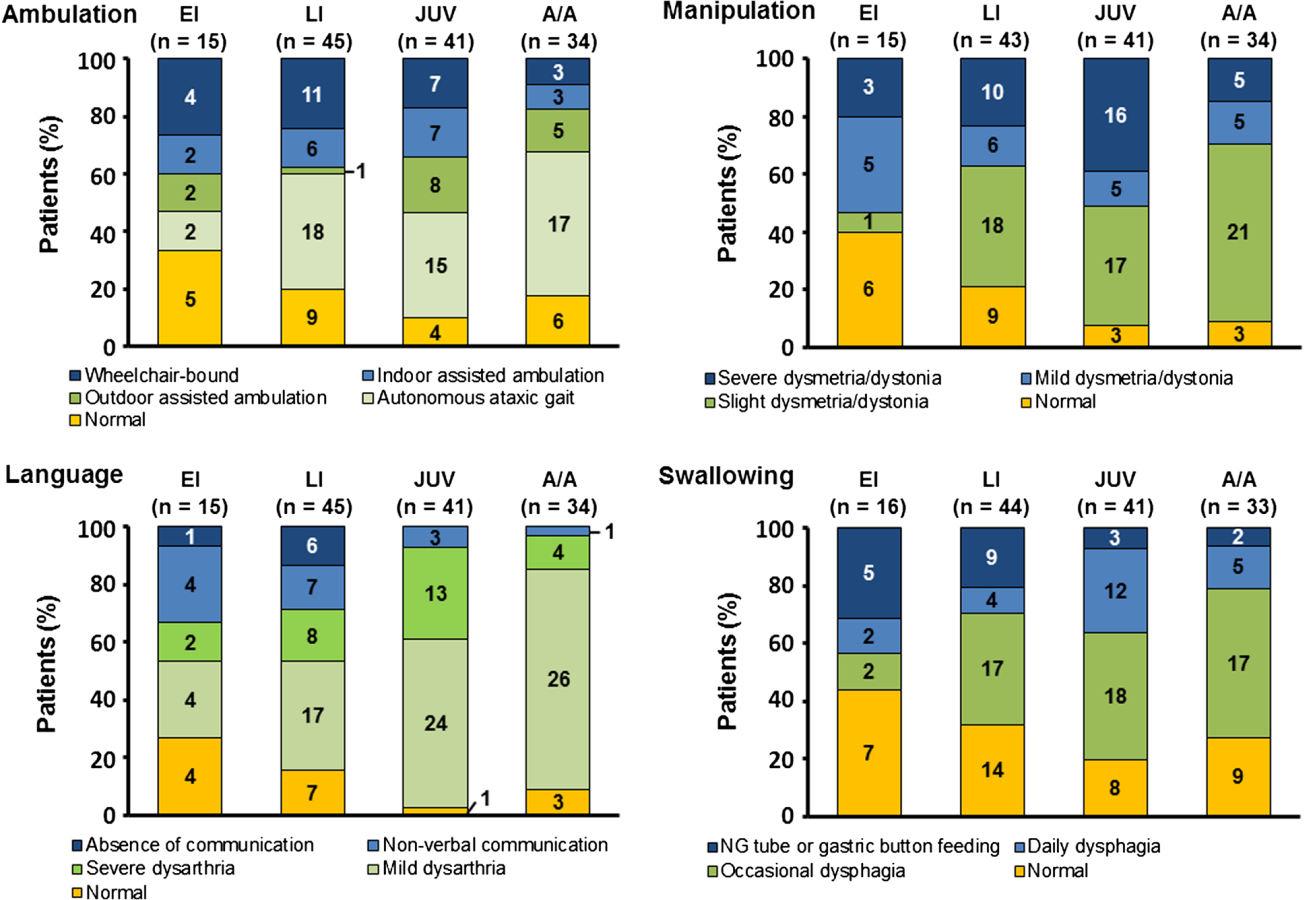
Disability scale scores by age of onset [EI = early infantile, LI = late infantile, JUV = juvenile, A/A = adolescent/adult].

A categorical analysis of scores for individual disability scale parameters by age at neurological onset group is summarised in Figure 4. Across all age-at-onset categories, more patients were categorised as having severe impairments in ambulation and manipulation than in language and swallowing. Severe impairments in language and swallowing were more common in early-infantile and late-infantile onset patients compared with juvenile and adolescent/adult-onset patients.

Among all patients, means (SD [95% CIs of the mean]) for individual disability domain scores were 0.42 (0.35 [0.36, 0.48]) for ambulation, 0.48 (0.35 [0.42, 0.54]) for manipulation, 0.35 (0.26 [0.31, 0.39]) for language and 0.37 (0.33 [0.32, 0.43]) for swallowing.

Mean (SD) composite disability scores were 0.40 (0.35) in early-infantile onset patients, 0.41 (0.30) in late-infantile onset patients, 0.47 in juvenile-onset patients and 0.35 (0.20) in adolescent/adult-onset patients. The 95% CIs of the mean scores in these patient groups overlapped across all age categories, indicating no notable differences between patients based on age at neurological onset (Table 2).

**Table 2:** Composite disability scores

**Patient group**	**n**	**Mean (SD)**	**95**% **CI of mean**	**Median (range)**
All patients with available scores	146	0.39 (0.28)	0.35, 0.44	0.35 (0–1.00)
Age at neurological onset*:				
Early infantile (<2 yrs)	14	0.40 (0.35)	0.19, 0.60	0.42 (0–1.00)
Late infantile (2 – <6 yrs)	42	0.41 (0.30)	0.32, 0.51	0.35 (0–1.00)
Juvenile (6 – <15 yrs)	41	0.47 (0.24)	0.39, 0.55	0.44 (0–0.88)
Adolescent/adult (≥15 yrs)	33	0.35 (0.20)	0.28, 0.43	0.29 (0.06–0.94)

*Age at neurological onset not available for 16 patients.

### Miglustat exposure

A total of 117/163 (72%) patients were recorded as being treated with miglustat therapy at enrolment. The mean (SD) duration of exposure to miglustat was 1.78 (1.88) years (median 1.13; range 0–7.73 years). Individual patient disease and treatment timelines indicated that the period from diagnosis to initiation of miglustat therapy was highly variable across all age-at-onset groups (Figure 1).

A total of 10 patients with neurological manifestations of NP-C were treated before having a confirmed diagnostic test result (excluding patients with missing test dates). Some cases were treated based on sibling disease history, and others were treated from the time physicians requested confirmatory diagnostic tests (already having a high suspicion for NP-C based on the overall clinical picture).

Four patients were treated before the onset of neurological manifestations, and at the time of enrolment in the Registry, three had developed neurological manifestations. One patient had early-infantile onset of neurological manifestations and was aged 0.8 years at miglustat start, two had late infantile-onset and were aged 2.1 and 5.3 years at miglustat start, and one had adolescent/adult-onset and was aged 16.3 years at miglustat start. The time period from the start of miglustat therapy to onset of neurological manifestations in these patients ranged from approximately 5 months to approximately 17 months. All four patients had confirmed diagnoses based on genetic testing; one patient also had a positive filipin staining result.

## Discussion

These observations come from an international registry population of 163 patients, which represents the largest cohort of living NP-C patients reported to date. As such, the findings reported in this paper provide an important contribution to existing knowledge regarding both disease and patient characteristics. This Registry population also comprises appreciable numbers of patients across all age-at-neurological-onset categories (early-infantile, late-infantile, juvenile and adolescent/adult). Notably, adolescent/adult onset patients make up almost one-third of the Registry population.

As has been reported in previous NP-C patient cohorts, a high proportion of early-infantile and late-infantile onset patients had a history of neonatal jaundice, hepatomegaly and/or splenomegaly [2,9]. This confirms the importance of these symptoms as tangible clues toward a possible diagnosis of NP-C in new-borns, although it should be borne in mind that they are often transient manifestations [11,12].

Interestingly, a notable proportion of adolescent/adult-onset patients in the NPC Registry also had a record of neonatal jaundice and/or hepatosplenomegaly during infancy. Despite memory bias, hepatosplenomegaly in older-onset patients often goes unrecognised [15]. Abdominal ultrasound examination and/or a detailed investigation of patients’ medical histories with regard to previous liver disease may help to detect systemic NP-C symptoms during diagnostic work up in adolescent/adult cases [10,22].

The profile of neurological symptoms in this Registry was consistent with data from previous large cohort studies [2,5,6,9]. In particular, VSGP was confirmed as a frequent finding across all age-at-onset categories. Saccadic eye movement abnormalities are known to be one of the earliest specific signs of neurological deterioration in NP-C, progressing over time to full VSGP in most cases [10,23,24]. These data therefore support existing knowledge and underline the importance of systematic examination of saccades and vergence movements as well as pursuit movements in the detection of possible NP-C [10,25-27].

The common occurrence of cataplexy and seizures in late-infantile and juvenile-onset NP-C patients, with lower levels seen in early-infantile and adolescent/adult-onset patients, is also in close agreement with existing reports [2,10]. These manifestations impair patient quality of life and in some severe cases, prognosis. Treatment directed toward achieving adequate control of cataplexy and seizures is essential [10].

Cognitive impairment was one of the most consistent neurological findings, reported in 60–70% of patients across all age-at-onset categories. While progressive cognitive decline eventually affects most, if not all, patients with NP-C, it is generally less commonly recognised during early childhood. Impaired cognition frequently manifests as poor school performance in juveniles and adolescents, and as NP-C progresses patients experience a general decline, leading to frank dementia in many cases [10,15,28,29]. In younger-onset patients it is more likely to be recognised initially as developmental delay [5].

Similar to cognitive decline, previous studies have reported psychiatric manifestations more frequently among adolescent/adult-onset NP-C patients compared with younger-onset patients [2,14,15]. In the Registry population 40% of adolescent/adult-onset patients had psychiatric manifestations, although this value might have been artificially high due to ‘cognitive decline’ having been recorded as a psychiatric manifestation; this was certainly the case in the early- and late-infantile groups. Nevertheless, schizophrenia-like psychosis has previously been reported in up to 25% of NP-C patients, and other major psychiatric illnesses reported in association with NP-C include depression, bipolar disorder and obsessive-compulsive disorder [10]. The frequency of NP-C in patients diagnosed with psychiatric disorders is an important aspect because in some patients, psychiatric manifestations can overshadow systemic symptoms and subtle neurological signs of NP-C, which leads to significant delays in the detection and diagnosis of NP-C [30,31]. Studies are currently ongoing to further establish the frequency of NP-C among psychiatric patients [32].

Analysis of patient age milestones illustrated that early-infantile patients have a greater tendency to be diagnosed before the onset of neurological manifestations compared with late-infantile, juvenile and adolescent/adult-onset patients, who are diagnosed after neurological onset. This is likely due to the greater prominence of systemic symptoms in very young patients. Overall, there were long delays between the onset of neurological manifestations and confirmation of the diagnosis of NP-C. This finding is consistent with data from previous studies [10,18,33], and highlights this major problem in NP-C.

Delayed diagnosis can have a considerable impact on quality of life for patients and/or their family and primary caregivers. In addition, it has been recommended that disease-specific therapy with miglustat should be started as early as possible in the course of neurological disease in order to stabilise neurological function [1,10]. Possible measures that might enhance the detection of NP-C and expedite future diagnoses include the use of novel screening tools such as the NP-C suspicion index [27], increased vigilance for early systemic symptoms, and the development of potential new disease biomarkers such as plasma oxysterols [34].

The composite disability scores among patients in the Registry at enrolment were comparable with baseline scores reported in previous cohort studies that utilised this disability scale [7,18,35]. It is notable that there were no significant differences in composite scores between age-at-onset categories among the Registry patients. The 95% CIs of the mean composite scores all overlapped. It also remains to be seen whether changes in composite scores over time in this Registry reflect previously observed differences in the rates of disease progression between the age-at-onset categories.

It is not possible to state whether any single disability scale measure (ambulation, manipulation, language or swallowing) was statistically more affected than others at enrolment based on the current categorical analysis. However, the distribution of scores for each disability parameter in the Registry appeared comparable with those seen at baseline during a previous cohort study based on the same disability scale [18].

The data summarised in this report should be interpreted with the awareness that disease registries come with a number of inherent limitations. As with all disease registries, the integrity and comparability of the NPC Registry data relies on the accurate entry of patient information by the treating physicians and other nominated staff at each participating centre. The potential for ascertainment bias (also present to some extent in all published NP-C cohorts) should be recognised. Registries can also suffer from selection bias. In the former case, the inclusion of all consecutive patients (both incident and prevalent) who present for outpatient and/or inpatient visits irrespective of treatment status is one measure stipulated in the NPC Registry protocol to minimise selection bias. Finally, most patients with a confirmed diagnosis of NP-C are currently being treated with miglustat, and there are only a few treatment-naïve patients available for study within the Registry population. At this point in time, this precludes meaningful comparisons between treated and untreated patients.

In summary, these data derived from the largest NP-C Registry cohort reported to date, provide a useful overall snapshot of patient and disease characteristics that can be considered alongside epidemiological data from previous large cohort studies. Our findings confirm that systemic symptoms are common among patients with early-childhood onset of neurological manifestations but are also a common finding among patients with adolescent/adult onset disease. The profiles of neurological manifestations seen among all age-at-onset categories in the Registry are in line with previous publications. Longitudinal data from follow up assessments are required to discern any differences in disease progression and degrees of neurological stability between early-infantile, late-infantile, juvenile and adolescent/adult-onset groups, and will be reported in future publications.

## Abbreviations

CI: Confidence interval; NP-C: Niemann-Pick disease type C; *NPC1*/*NPC2* mutations: Disease-causing gene mutations in NP-C; SD: Standard deviation; VSGP: Vertical supranuclear gaze palsy.

## Competing interests

MCP has received research grants from the National Institutes of Health [NS 65768–01], the National MS Society and Actelion Pharmaceuticals Ltd . He has also received honoraria and consulting fees from Actelion Pharmaceuticals Ltd, Orphazyme, Shire HGT, Stem Cells, Inc, and Up-To-Date. EM has received consulting fees, honoraria and research grants from Actelion Pharmaceuticals Ltd. FAW has received consulting fees, honoraria and research grants from Actelion Pharmaceuticals Ltd. AM and BS are permanent employees of Actelion Pharmaceuticals Ltd. HD (Numerus Ltd) conducted statistical analyses that were paid for by Actelion Pharmaceuticals Ltd. MTV has received travel expenses, consulting fees and presentation honoraria from Actelion Pharmaceuticals Ltd and Shire HGT, and travel expenses from Genzyme Corporation. MP has received consulting fees, honoraria and research grants from Actelion Pharmaceuticals Ltd.

## Authors’ contributions

All authors have reviewed and interpreted the data, reviewed each draft of the manuscript, and approved the final version for submission.
