# Correction: Disease and patient characteristics in NP-C patients: findings from an international disease registry

**DOI:** 10.1186/1750-1172-8-73

**Published:** 2013-05-14

**Authors:** Marc C Patterson, Eugen Mengel, Frits A Wijburg, Audrey Muller, Barbara Schwierin, Harir Drevon, Marie T Vanier, Mercé Pineda

**Affiliations:** 1 Mayo Clinic, Rochester, MN, USA; 2 Villa Metabolica, ZKJM, MC, University of Mainz,, Mainz, Germany; 3 Academic Medical Centre, University of Amsterdam, Amsterdam, The Netherlands; 4 Actelion Pharmaceuticals Ltd, Allschwil, Switzerland; 5 Numerus Ltd, Wokingham, UK; 6 INSERM Unit 820, Lyon, France; 7 Fundació Hospital Sant Joan de Déu, Barcelona, Spain; 8 Department of Neurology, Mayo Clinic, 200 First Street SW, Rochester, MN, 55905, USA

## Correction

After the publication of this work [1] it was brought to the authors attention that Figure 1 contained an inversion in the color of the triangles, where yellow which should be "diagnosis" is said to be "first miglustat use", and blue which should be " first miglustat use" is said to be " diagnosis". The correct figure is given below:

**Figure 1:**
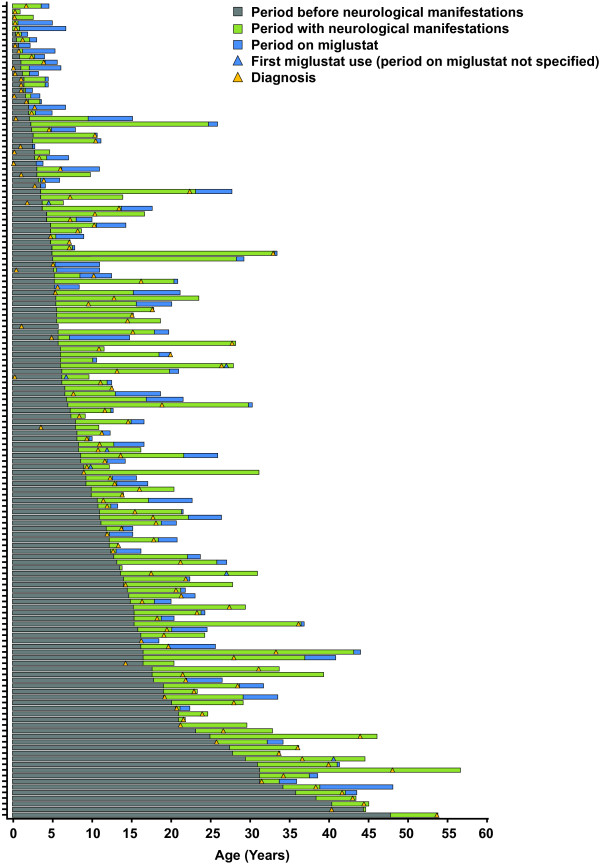
**Overview of patient and disease characteristics.** We regret any inconvenience that this inaccuracy may have caused.
